# Heavy Lifetime Cannabis Use and Mortality by Sex

**DOI:** 10.1001/jamanetworkopen.2024.15227

**Published:** 2024-06-06

**Authors:** Alexandre Vallée

**Affiliations:** 1 Department of Epidemiology and Public Health, Foch Hospital, Suresnes, France

## Abstract

**Importance:**

The association between mortality and cannabis use remains unclear.

**Objective:**

To examine sex-stratified associations of cumulative lifetime cannabis use with all-cause, cardiovascular disease (CVD), and cancer mortality in the UK Biobank population.

**Design, Setting, and Participants:**

This cohort study used data from volunteers in the UK Biobank population. Participant monitoring for mortality in the UK Biobank study commenced from the point of their inclusion between 2006 and 2010 and continued until December 19, 2020. Data regarding the causes of death were sourced from the National Health Service Information Centre. Data were analyzed from inception of study inclusion to December 2020.

**Exposure:**

Cannabis use status was assessed by questionnaire and categorized as heavy, moderate, low, and never.

**Main Outcomes and Measures:**

The main outcomes were all-cause, CVD, and cancer mortality. Sex-stratified associations of cumulative lifetime cannabis use with mortality were estimated using Cox proportional hazards regression with adjustment for demographic and clinical variables.

**Results:**

Among 121 895 participants (54.51% females with mean [SD] age of 55.15 [7.64] years; 45.49% males with mean [SD] age of 56.46 [7.79] years) during an overall median of 11.80 years (IQR, 10.53-13.22 years) of follow-up, 2375 total deaths occurred, including 1411 deaths from CVD and 440 from cancer. In males, after full adjustment, the hazard ratios (HRs) were 1.28 (95% CI, 0.90-1.81) for all-cause mortality, 0.98 (95% CI, 0.43-2.25) for CVD mortality, and 1.09 (95% CI, 0.71-1.67) for cancer mortality among heavy cannabis users compared with never users. In females, after full adjustment, the HRs were 1.49 (95% CI, 0.92-2.40) for all-cause mortality, 2.67 (95% CI, 1.19-4.32) for CVD mortality, and 1.61 (95% CI, 0.91-2.83) for cancer mortality among heavy cannabis users compared with never users. In female current tobacco users, after full adjustment, heavy cannabis use was associated with all-cause mortality (HR, 2.25; 95% CI, 1.12-4.53), CVD mortality (HR, 2.56; 95% CI, 1.43-15.36), and cancer mortality (HR, 3.52; 95% CI, 1.50-8.33) and among never tobacco users was associated with CVD mortality (HR, 2.98; 95% CI, 1.67-6.61). In male current tobacco users, heavy cannabis use was associated with cancer mortality (HR, 2.44; 95% CI, 1.14-5.23).

**Conclusions and Relevance:**

In this study, a positive association between CVD mortality and heavy lifetime cannabis use was observed among females. Longitudinal studies are needed in general populations to investigate the potential effects of cannabis on mortality.

## Key Points

**Question:**

Is there an association between cannabis use and all-cause, cardiovascular disease (CVD), and cancer mortality?

**Findings:**

In this cohort study of 121 895 participants, in the fully adjusted model among females, the risk for CVD mortality was significantly higher among heavy cannabis users compared with never users; there was no association among males. No association was observed among females or males for all-cause and cancer mortality.

**Meaning:**

The findings suggest that heavy cannabis use is associated with CVD mortality among females.

## Teaser

This cohort study of data from the UK Biobank examines associations between cumulative lifetime cannabis use and all-cause, cardiovascular disease, and cancer mortality.

## Introduction

Cannabis is the most commonly consumed illegal drug globally. Considering the growing movement toward legalizing cannabis across various regions, it has become increasingly critical to comprehend the health consequences of its habitual use. Recent research has indicated a potential increase in cardiovascular (CV) risks associated with cannabis consumption.^1,2,3,4,5^ However, these studies have been constrained by their focus on specific population segments, which may affect the perceived association of cannabis with CV health.^6,7^ Additionally, there has been a scarcity of research exploring how cannabis use might affect females and males differently.^8,9^ Alongside these concerns, the use of cannabis for medical purposes is rapidly expanding,^10^ yet our understanding of its safety and effectiveness for different medical conditions remains limited.^11^

While the use of cannabis continues to gain popularity, its broader effects on public health within the general population is not yet fully understood. It was acknowledged in 1 study^12^ that using cannabis is associated with a heightened risk of being involved in motor vehicle accidents. Additionally, emerging research suggests a possible association between cannabis use and the onset of acute myocardial infarction as well as an elevated risk of ischemic stroke.^13^ Yet, when it comes to understanding how cannabis use might relate to overall mortality and specific causes of death, the research landscape is limited and presents conflicting findings.

To date, only a couple of studies (of Swedish men aged 18-19 years^14^ and US patients aged 40 to 49 years^15^) have suggested that heavy use of marijuana may be associated with an increased risk of death from all causes,^14,15^ and only 1 study suggested an association with CV mortality.^16^ On the other hand, several other studies have not found any association between marijuana use and mortality from all causes or specifically from CV diseases (CVDs).^7,17,18,19^ It is important to note, however, that those studies’ conclusions are potentially limited by factors such as varying methods of measuring marijuana exposure, a limited age range of participants (15-50 years for most of the studies), small sample sizes, or short follow-up durations.^7,14,15,17,18,19^ Furthermore, to my knowledge, only 1 study thus far has explored the association between marijuana use and cancer mortality, but it did not find an association.^16^

In 2018, the 12-month prevalence of cannabis use was estimated at 3.9% worldwide; 15.4% of European Union inhabitants aged 15 to 35 years used cannabis in the previous year, and 0.6% of the European population met criteria for cannabis use disorder, resulting in 158 million disability-adjusted life-years.^20^ Thus, a necessity to understand the association between cannabis use and mortality risk has become more pressing. There is a crucial need to assess the risks of all-cause mortality and its specific underlying causes in association with cannabis use among general populations. The purpose of this study was to examine sex-stratified associations of cumulative lifetime cannabis use with all-cause, CVD, and cancer mortality in the UK Biobank population.

## Methods

### UK Biobank Population

This cohort study used data from the UK Biobank. The UK Biobank is a forward-looking cohort initiative aimed at investigating, preventing, diagnosing, and treating chronic diseases—notably CVD—in adults. The extensive study encompassed 502 478 individuals from across 22 cities in the UK, all registered with the UK National Health Service. Participants were aged between 40 and 69 years when they joined the UK Biobank between 2006 and 2010 and were thoroughly phenotyped and genotyped. This process involved responses to detailed questionnaires, participation in computer-assisted interviews, and undergoing various physical and functional assessments. Additionally, the collection of biological samples, including blood, urine, and saliva, was integral to the study.^21^ All participants in the UK Biobank study provided their informed consent electronically. The UK Biobank obtained ethical approval from the North West Multi-center Research Ethics Committee, which extends its jurisdiction across the entire UK. The current study adhered to the principles outlined in the Declaration of Helsinki^22^ and received approval from the North West–Haydock Research Ethics Committee. This study followed the Strengthening the Reporting of Observational Studies in Epidemiology (STROBE) reporting guideline.

The data gathered in the UK Biobank encompass a wide range of factors. These include socioeconomic variables, behavioral and lifestyle information, a comprehensive mental health assessment, clinical diagnoses, and treatments. Furthermore, the cohort has been a rich source of genetic information, with imaging and physiological biomarkers obtained from blood and urine samples. The protocol that guided this extensive collection and analysis of data are thoroughly documented in the scientific literature.^23^

### Ascertainment of Mortality

Participant monitoring for mortality in the UK Biobank study commenced from the point of their inclusion. Follow-up was systematically concluded on December 19, 2020, for all participants. Data regarding the causes of death were sourced from the National Health Service Information Centre. For those interested in the specifics of how these data were linked and processed, comprehensive details are accessible online.^24^

Causes of CVD mortality were defined with main cause of death corresponding to *International Statistical Classification of Diseases and Related Health Problems, Tenth Revision (ICD-10)* codes I00 to I78, G951, H341, H342, O10, S066, Z951, and Z955.^25^ Causes of cancer mortality were defined with main cause of death corresponding to *ICD-10* codes C00 to C96, D00 to D48, and Z85.^25^

### Cannabis Use

Cannabis use was determined through a self-reported questionnaire. Participants were queried about their total lifetime use of cannabis with the question, “Have you ever used cannabis (marijuana, grass, hash, ganja, blow, draw, skunk, weed, spliff, dope), even if it was a long time ago?” Based on their responses, those who answered “no” were categorized as control individuals, while those who answered “yes” were classified as cannabis users. Furthermore, I divided these users into 3 distinct groups, reflecting the frequency of their reported use per the questionnaire categories. These categories included low users of cannabis, defined as those who reported lifetime use 1 to 2 times or 3 to 10 times, and continued users, subdivided into moderate users (11-100 times) and heavy users (>100 times).^26^ Heavy cannabis use could also be defined, as in the literature, as daily or near daily use for at least a few months.^27^

### Covariates

Systolic and diastolic blood pressure were measured twice at the assessment center during the inclusion consultation using an automated blood pressure device (OMRON 705IT electronic blood pressure monitor [OMRON Healthcare Europe]). Alternatively, measurements were taken manually with a sphygmomanometer and stethoscope in cases in which the blood pressure device failed or the largest cuff did not fit the individual’s arm.^28^

Hypertension was defined as having a systolic blood pressure of at least 140 mm Hg and/or a diastolic blood pressure of at least 90 mm Hg. This definition aligns with the guidelines set by the European Society of Cardiology and includes cases managed with antihypertensive drugs or diagnosed by a clinician.

Diabetes status was determined by either the use of antidiabetic medication, a diagnosis of diabetes, or a fasting glucose concentration of 126.13 mg/dL or greater (to convert to mmol/L, multiply by 0.0555).^29^ Dyslipidemia was identified if an individual had a fasting plasma total cholesterol level of 255.21 mg/dL or greater, low-density lipoprotein cholesterol level of 158.30 mg/dL or greater (to convert to mmol/L, multiply by 0.0259), or triglyceride level greater than 150.44 mg/dL (to convert to mmol/L, multiply by 0.0113) or if they were taking statin medication. Medication use was assessed through the question, “Do you regularly take any of the following medications?” Cardiovascular diseases were defined by myocardial infarction, angina, and stroke as diagnosed by a clinician and reported in questionnaires.

Body mass index (BMI) was calculated as weight in kilograms divided by height in meters squared and was categorized as high (>30), moderate (25-30), or low (<25). Detailed information on biological parameters can be found in the UK Biobank protocol.^30^

Educational level was classified into 3 categories: high (college or university degree), intermediate (A/AS levels or equivalent, O levels/General Certificate of Secondary Education or equivalent, or other professional qualifications [eg, nursing, teaching]), and low (none of the aforementioned). Income level was categorized as high (>£52 000 [US$65 312] per year), moderate (£18 000-£51 999 [US$22 608-$65 311] per year), and low (<£18 000 [US$22 608] per year).

Tobacco smoking and alcohol use were self-reported. For alcohol use status, participants had to report their alcohol use as “current,” “past,” or “never.” In the UK Biobank, the number of years of smoking is calculated by subtracting the age of starting smoking from the age smoking was stopped (or age at inclusion for current tobacco users) using the following equation: pack-years = number of cigarettes per day/(20 × [age stopped smoking − age started smoking]). Antidepressant use was reported by Davis et al^31^ and was included as a confounding factor due to its association with CV risk^32^ and the association between cannabis use and depression.^33^

### Statistical Analysis

Characteristics of the study population were described as means with SDs for continuous variables. Categorical variables were described as numbers and proportions. Comparisons between groups were performed using *t* tests for continuous variables; analysis of variance was performed to assess differences between groups. Pearson χ^2^ test was performed for categorical variables. Statistical analyses were stratified by sex due to cannabis consumption differences between females and males.^34,35^

Cox proportional hazards regression models were used to estimate the hazard ratios (HRs) and 95% CIs for the associations between cannabis use and risk of all-cause mortality and cause-specific hazard models for the risk of cause-specific mortality (CV and cancer). Kaplan-Meier analyses censored at 10 years’ follow-up were performed and compared by log-rank test. Follow-up time for each participant was calculated as the difference between the examination date in the UK Biobank and the last known date alive (December 19, 2020) or censored from the linked mortality-life data.

First, sex-stratified Cox proportional hazards regression models were adjusted for age. Second, the fully sex-stratified Cox proportional hazards regression models were adjusted for age, educational level, income, smoking history (pack-years), alcohol status, hypertension, diabetes, dyslipidemia, BMI, previous CVDs, and antidepressant medication use. Never users of cannabis were considered as the reference group in the analyses. Statistical analyses were performed from inception of study inclusion to December 2020 using SAS software, version 9.4 (SAS Institute Inc). Two-sided *P* <.05 was considered statistically significant.

## Results

**Figure 1.**
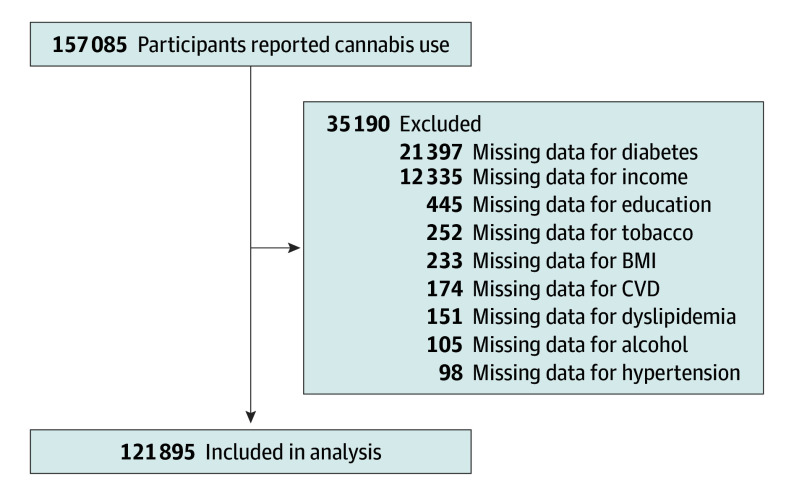
Flowchart of Study Participants BMI indicates body mass index; CVD, cardiovascular disease.

A total of 157 085 volunteers in the UK Biobank study who responded to the questions of cannabis use were included. Of them, 35 190 were excluded for missing data. Therefore, the study analyzed 121 895 volunteers (Figure 1): 66 444 females (54.51%) with mean (SD) age of 55.15 (7.64) years and 55 451 males (45.49%) with mean (SD) age of 56.46 (7.79) years. A total of 2154 males (3.88%) were heavy cannabis users compared with 1287 females (1.94%). Heavy cannabis users were more likely to be younger, report tobacco use, and show lower levels of alcohol use, hypertension, dyslipidemia, obesity, diabetes, high education, and high income (Table 1).

**Table 1.** Characteristics of the Study Population According to Sex and Cannabis Use Status, 2006-2010

Characteristic	Participants (N = 121 895)^a^									
	Female (n = 66 444)					Male (n = 55 451)				
	Heavy users (n = 1287)	Moderate users (n = 2703)	Low users (n = 9773)	Never users (n = 52 681)	*P* value	Heavy users (n = 2154)	Moderate users (n = 3019)	Low users (n = 9293)	Never users (n = 40 985)	*P* value
Age, mean (SD), y	50.52 (6.76)	51.28 (6.83)	52.51 (7.22)	55.95 (7.56)	<.001	50.92 (6.99)	52.00 (7.06)	53.92 (7.47)	57.66 (7.56)	<.001
Follow-up, mean (SD), y	11.73 (0.89)	11.79 (0.89)	11.78 (0.88)	11.82 (0.87)	<.001	11.76 (0.90)	11.75 (0.89)	11.80 (0.89)	11.80 (0.90)	.001
Age at death, mean (SD), y	84.62 (3.05)	84.76 (2.37)	84.80 (1.92)	84.79 (1.90)	.04	84.62 (2.86)	84.70 (2.46)	84.74 (2.07)	84.65 (2.25)	.003
CVD	13 (1.01)	31 (1.15)	107 (1.09)	1027 (1.95)	<.001	67 (3.11)	108 (3.58)	397 (4.27)	2591 (6.32)	<.001
Hypertension	240 (18.65)	560 (20.72)	2444 (25.01)	18 039 (34.24)	<.001	874 (40.58)	1317 (43.62)	4546 (48.92)	23 545 (57.45)	<.001
Diabetes	37 (2.87)	100 (3.70)	317 (3.24)	2196 (4.17)	<.001	107 (4.97)	136 (4.50)	537 (5.78)	2856 (6.97)	<.001
Dyslipidemia	502 (39.01)	951 (35.18)	3816 (39.05)	24 874 (47.22)	<.001	1268 (58.87)	1756 (58.16)	5642 (60.71)	25 961 (63.34)	<.001
Alcohol use										
Current	1230 (95.57)	2619 (96.89)	9449 (96.68)	49 052 (93.11)	<.001	2012 (93.41)	2918 (96.65)	9054 (97.43)	39 046 (95.27)	<.001
Past	47 (3.65)	67 (2.48)	257 (2.63)	1455 (2.76)		133 (6.17)	96 (3.18)	214 (2.30)	992 (2.42)	
Never	10 (0.78)	17 (0.63)	67 (0.69)	2174 (4.13)		9 (0.42)	5 (0.17)	25 (0.27)	947 (2.31)	
Educational level^b^										
High	811 (63.01)	1860 (68.81)	6016 (61.56)	21 787 (41.36)	<.001	1231 (57.15)	1911 (63.30)	5516 (59.36)	18 304 (44.66)	<.001
Moderate	418 (32.48)	769 (28.45)	3370 (34.48)	25 123 (47.69)		779 (36.17)	931 (30.84)	3150 (33.90)	17 904 (43.68)	
Low	58 (4.51)	74 (2.74)	387 (3.96)	5771 (10.95)		144 (6.69)	177 (5.86)	627 (6.75)	4777 (11.66)	
Income^c^										
High	425 (33.02)	1119 (41.40)	4029 (41.23)	15 154 (28.77)	<.001	886 (41.13)	1569 (51.97)	4432 (47.69)	13 751 (33.55)	<.001
Moderate	625 (48.56)	1288 (47.65)	4655 (47.63)	28 746 (54.57)		973 (45.17)	1182 (39.15)	4074 (43.84)	22 203 (54.17)	
Low	237 (18.41)	296 (10.95)	1089 (11.14)	8781 (16.67)		295 (13.70)	268 (8.88)	787 (8.47)	5031 (12.28)	
Tobacco smoking										
Current	454 (35.28)	436 (16.13)	1094 (11.19)	2229 (4.23)	<.001	754 (35.00)	480 (15.90)	1174 (12.63)	2360 (5.76)	<.001
Past	755 (58.66)	1798 (66.52)	4994 (51.10)	14 283 (27.11)		1197 (55.57)	1757 (58.20)	4448 (47.86)	14 031 (34.23)	
Never	78 (6.06)	469 (17.35)	3685 (37.71)	36 169 (68.66)		203 (9.42)	782 (25.90)	3671 (39.50)	24 594 (60.01)	
Smoking history, mean (SD), pack-years	16 (13)	12 (13)	9 (13)	4 (10)	<.001	18 (16)	13 (16)	11 (16)	7 (14)	<.001
BMI^d^										
High	179 (13.91)	407 (15.06)	1625 (16.63)	10 412 (19.76)	<.001	350 (16.25)	549 (18.18)	1891 (20.35)	8950 (21.84)	<.001
Moderate	428 (33.26)	789 (29.19)	3245 (33.20)	18 779 (35.65)		990 (45.96)	1429 (47.33)	4561 (49.08)	20 430 (49.85)	
Low	680 (52.84)	1507 (55.75)	4903 (50.17)	23 490 (44.59)		814 (37.79)	1041 (34.48)	2841 (30.57)	11 605 (28.32)	
Antidepressant use	112 (8.70)	200 (7.40)	762 (7.80)	3895 (7.39)	.20	121 (5.62)	112 (3.71)	343 (3.69)	1481 (3.61)	<.001

Abbreviations: BMI, body mass index (calculated as weight in kilograms divided by height in meters squared); CVD, cardiovascular disease.

^a^ Data are presented as number (percentage) of participants unless otherwise indicated.

^b^ High educational level, college or university degree; moderate, A/AS levels or equivalent, O levels/General Certificate of Secondary Education or equivalent, or other professional qualifications; and low, none of these.

^c^ High income, greater than £52 000 (US$65 312) per year; moderate, £18 000 to £51 999 (US$22 608-$65 311) per year; and low, less than £18 000 (US$22 608) per year.

^d^ High BMI, greater than 30; moderate, 25 to 30; and low, less than 25.

**Figure 2.**
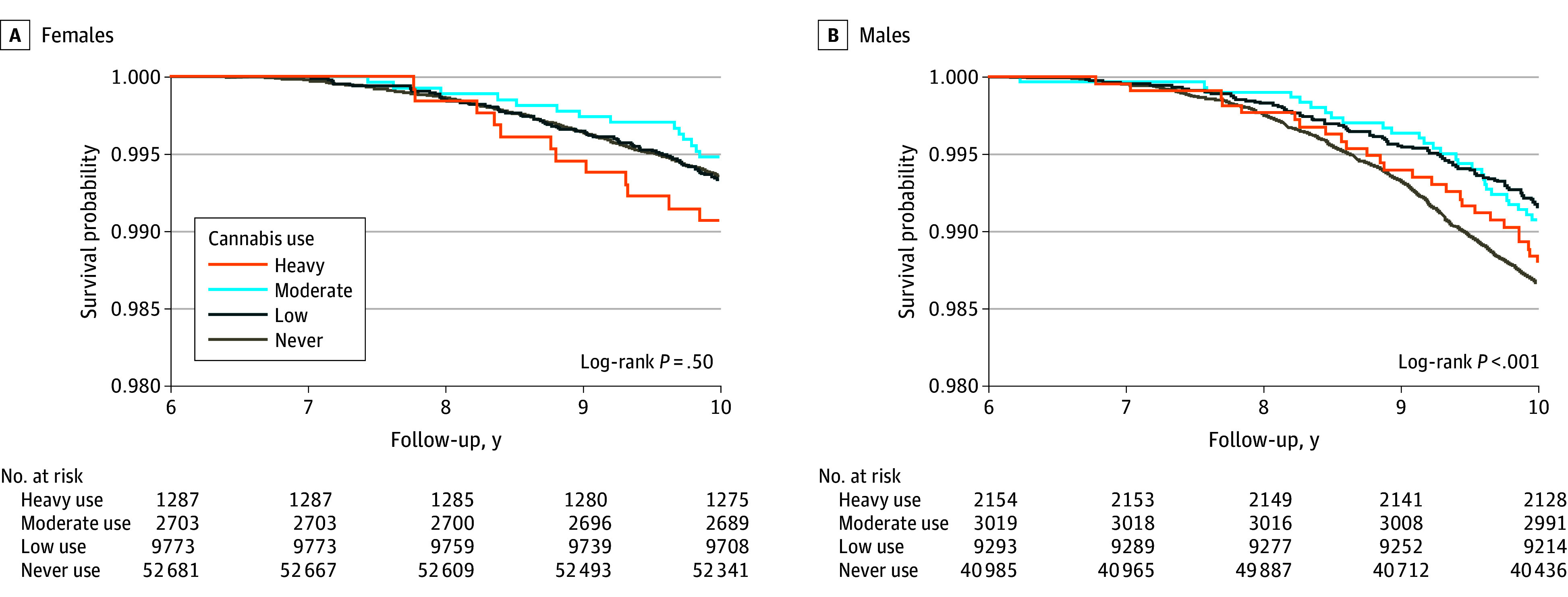
Kaplan-Meier Curves for All-Cause Mortality in Females and Males According to Cannabis Use

The median follow-up overall was 11.80 years (IQR, 10.53-13.22 years); among females, median follow-up was 11.81 years (IQR, 10.54-13.24 years), and among males, it was 11.80 years (IQR, 10.53-13.21 years). During this time, 2375 total deaths occurred, including 1411 deaths from CVD and 440 from cancer. A total of 964 deaths among females (1.45%) and 1401 deaths among males (2.53%) were from all causes, including 125 (0.19%) and 315 (0.57%) deaths from CVD, respectively, and 658 (0.99%) and 753 (1.36%) deaths from cancer, respectively. Figure 2 presents the evolution of death according to cannabis status in both sexes.

In males, after adjustment for age, the HRs among participants who were heavy cannabis users compared with never users were 1.50 (95% CI, 1.09-2.05) for all-cause mortality, 1.36 (95% CI, 0.70-2.69) for CVD mortality, and 1.18 (95% CI, 0.73-1.89) for cancer mortality (Table 2). In the fully adjusted model for males, the HRs among participants who were heavy cannabis users were 1.28 (95% CI, 0.90-1.81) for all-cause mortality, 0.98 (95% CI, 0.43-2.25) for CVD mortality, and 1.09 (95% CI, 0.71-1.67) for cancer mortality.

**Table 2.** Sex-Stratified Cox Proportional Hazards Regression Models for All-Cause, CVD, and Cancer Mortality

Model, variable	Hazard ratio (95% CI)		
	All-cause mortality	CVD mortality	Cancer mortality
**Females**			
Age-adjusted model			
Cannabis use			
Heavy	1.88 (1.21-2.91)	2.47 (1.13-5.38)	1.99 (1.21-3.29)
Moderate	1.20 (0.84-1.73)	2.32 (0.79-6.05)	1.10 (0.70-1.72)
Low	1.17 (0.96-1.41)	1.07 (0.60-1.92)	1.22 (0.97-1.53)
Never	1 [Reference]	1 [Reference]	1 [Reference]
Fully adjusted model^a^			
Cannabis use			
Heavy	1.49 (0.92-2.40)	2.67 (1.19-4.32)	1.61 (0.91-2.83)
Moderate	1.07 (0.72-1.60)	2.24 (0.68-3.32)	0.92 (0.55-1.56)
Low	1.07 (0.87-1.33)	0.94 (0.48-1.84)	1.20 (0.93-1.55)
Never	1 [Reference]	1 [Reference]	1 [Reference]
**Males**			
Age-adjusted model			
Cannabis use			
Heavy	1.50 (1.09-2.05)	1.36 (0.70-2.69)	1.18 (0.73-1.89)
Moderate	1.11 (0.83-1.48)	0.77 (0.38-1.58)	1.29 (0.89-1.86)
Low	0.95 (0.80-1.12)	0.76 (0.52-1.11)	1.03 (0.83-1.29)
Never	1 [Reference]	1 [Reference]	1 [Reference]
Fully adjusted model^a^			
Cannabis use			
Heavy	1.28 (0.90-1.81)	0.98 (0.43-2.25)	1.09 (0.71-1.67)
Moderate	0.97 (0.70-1.36)	0.80 (0.37-1.72)	1.01 (0.60-1.71)
Low	0.88 (0.72-1.07)	0.75 (0.49-1.14)	0.95 (0.74-1.21)
Never	1 [Reference]	1 [Reference]	1 [Reference]

Abbreviation: CVD, cardiovascular disease.

^a^ Adjusted for age, educational level, income, smoking history (pack-years), alcohol status, hypertension, diabetes, dyslipidemia, body mass index, previous CVDs, and antidepressant medication.

In females, after adjustment for age, the HRs among participants who were heavy cannabis users compared with never users were 1.88 (95% CI, 1.21-2.91) for all-cause mortality, 2.47 (95% CI, 1.13-5.38) for CVD mortality, and 1.99 (95% CI, 1.21-3.29) for cancer mortality (Table 2). In the fully adjusted model for females, the HRs among participants who were heavy cannabis users were 1.49 (95% CI, 0.92-2.40) for all-cause mortality, 2.67 (95% CI, 1.19-4.32) for CVD mortality, and 1.61 (95% CI, 0.91-2.83) for cancer mortality. Results for all covariates are shown in eTables 1 and 2 in Supplement 1.

In males who were current tobacco users, after adjustment for all covariates, heavy cannabis use was significantly associated with cancer mortality (HR, 2.44; 95% CI, 1.14-5.23). In females who currently used tobacco, after adjustment for all covariates, heavy cannabis use was significantly associated with all-cause mortality (HR, 2.25; 95% CI, 1.12-4.53), CVD mortality (HR, 2.56; 95% CI, 1.43-15.36), and cancer mortality (HR, 3.52; 95% CI, 1.50-8.33). Heavy cannabis use was associated with CVD mortality (HR, 2.98; 95% CI, 1.67-6.61) among females who had never used tobacco (Table 3).

**Table 3.** Fully Adjusted Sex-Stratified Cox Proportional Hazards Regression Models for All-Cause, CVD, and Cancer Mortality According to Tobacco Smoking Status

Variable	Hazard ratio (95% CI)		
	All-cause mortality	CVD mortality	Cancer mortality
**Females**			
Current tobacco use			
Cannabis use			
Heavy	2.25 (1.12-4.53)	2.56 (1.43-15.36)	3.52 (1.50-8.33)
Moderate	1.57 (0.68-3.63)	2.31 (0.39-13.56)	2.02 (1.04-3.92)
Low	1.59 (0.96-2.66)	0.62 (0.07-5.72)	2.10 (0.69-4.39)
Never	1 [Reference]	1 [Reference]	1 [Reference]
Past tobacco use			
Cannabis use			
Heavy	0.87 (0.38-1.99)	NA	1.03 (0.41-2.59)
Moderate	0.87 (0.49-1.57)	2.96 (0.92-9.53)	0.66 (0.30-1.45)
Low	1.09 (0.79-1.53)	1.32 (0.51-3.43)	1.27 (0.87-1.87)
Never	1 [Reference]	1 [Reference]	1 [Reference]
Never tobacco use			
Cannabis use			
Heavy	1.68 (0.23-11.97)	2.98 (1.67-6.61)	NA
Moderate	1.43 (0.64-3.22)	2.39 (0.32-7.59)	1.33 (0.49-3.59)
Low	0.81 (0.56-1.21)	0.87 (0.27-2.82)	0.86 (0.54-1.34)
Never	1 [Reference]	1 [Reference]	1 [Reference]
**Males**			
Current tobacco use			
Cannabis use			
Heavy	1.51 (0.83-2.73)	1.11 (0.32-3.89)	2.44 (1.14-5.23)
Moderate	1.15 (0.64-2.05)	0.90 (0.22-2.92)	1.14 (0.46-2.82)
Low	1.09 (0.71-1.68)	0.53 (0.18-1.58)	1.65 (0.94-2.91)
Never	1 [Reference]	1 [Reference]	1 [Reference]
Past tobacco use			
Cannabis use			
Heavy	1.25 (0.75-2.08)	1.10 (0.33-3.63)	1.15 (0.57-2.32)
Moderate	0.78 (0.46-1.30)	0.68 (0.21-2.20)	0.83 (0.43-1.62)
Low	0.95 (0.72-1.25)	0.83 (0.46-1.52)	0.99 (0.68-1.44)
Never	1 [Reference]	1 [Reference]	1 [Reference]
Never tobacco use			
Cannabis use			
Heavy	0.85 (0.21-3.43)	NA	NA
Moderate	0.85 (0.40-1.80)	0.56 (0.07-4.07)	0.78 (0.29-2.12)
Low	0.83 (0.43-1.91)	0.71 (0.35-1.48)	0.61 (0.28-1.99)
Never	1 [Reference]	1 [Reference]	1 [Reference]

Abbreviations: CVD, cardiovascular disease; NA, not applicable.

When excluding participants with hypertension, diabetes, obesity, current tobacco use, and previous CVD (55 517 participants [45.54%]), in females, heavy cannabis use was not associated with all-cause mortality (HR, 1.48; 95% CI, 0.64-3.39), cancer mortality (HR, 1.81; 95% CI, 0.73-4.50), or CVD mortality (HR, 1.81; 95% CI, 0.39-8.34). Similar results were observed among males (all-cause: HR, 1.15 [95% CI, 0.50-2.65]; cancer: HR, 0.92 [95% CI, 0.29-2.95]; CVD: HR, 1.35 [95% CI, 0.85-6.74]).

Moreover, in stratified analyses, we found that among females with overweight, heavy cannabis use was associated with all-cause mortality (HR, 2.23; 95% CI, 1.11-4.45) and cancer mortality (HR, 2.79; 95% CI, 1.32-5.88). Among males without hypertension, heavy cannabis use was associated with all-cause mortality (HR, 1.52; 95% CI, 1.01-2.32). Heavy cannabis use was associated with all-cause mortality (HR, 2.14; 95% CI, 1.29-3.54) and cancer mortality (HR, 2.43; 95% CI, 1.36-4.32) among females without hypertension. Among females without diabetes, heavy cannabis use was associated with CVD mortality (HR, 2.92; 95% CI, 1.21-7.05) (eTables 3-5 in Supplement 1).

When considering only the status of cannabis use among users compared with never users, no significant association with all-cause mortality (HR, 1.11; 95% CI, 0.91-1.34), CVD mortality (HR, 1.35; 95% CI, 0.80-2.27), or cancer mortality (HR, 1.18; 95% CI, 0.94-1.49) was shown among females. Similar results were observed among males (all-cause: HR, 1.06 [95% CI, 0.90-1.24]; CVD: HR, 1.27 [95% CI, 0.89-1.82]; cancer: HR, 1.02 [95% CI, 0.82-1.26]).

## Discussion

The relationship between cannabis use and specific causes of mortality in the general population remains largely unexplored. Previous research has primarily focused on all-cause mortality among younger groups,^14,15,16,17,18,19^ with the majority of these studies,^14,15,18,19^ but not all,^16,17^ indicating a heightened risk of all-cause mortality associated with cannabis use. However, only a handful of studies have delved into the potential association between cannabis use and CVD mortality but found no association^15,17,19^; an exception is a study focused on 14 818 middle-aged (age 20-59 years) US adults with a median follow-up of 5.8 years that showed a significant association between ever use of cannabis and CVD mortality (HR, 2.29; 95% CI, 1.10-4.78).^16^ In contrast, another study tracking 1913 adults hospitalized for myocardial infarction over a median of 3.8 years reported a nondefinitive adjusted HR of 1.9 (95% CI, 0.6-6.3) for CVD mortality.^15^ This aligns with existing research indicating a greater risk of acute myocardial infarction and ischemic stroke associated with cannabis use.^2,36^ Other studies on this topic that primarily involved younger participants aged 18 to 30 years^19^ and 15 to 49 years^17^ did not find that higher mortality was associated with cannabis use.

The potential connection between cannabis use and CVD mortality is biologically credible. Research suggests that certain cannabinoids, like D9-tetrahydrocannabinol, that are key components of cannabis may contribute to inflammation, endothelial dysfunction, and atherosclerosis development.^37^ Another contributing factor could be the increased exposure to carbon monoxide through cannabis smoking, leading to higher levels of carboxyhemoglobin in the blood.^38^ This may impair the oxygen-carrying capacity in blood, reducing oxygen availability in tissues and cells, including myocardial cells, and potentially triggering angina, especially in individuals with preexisting coronary heart disease.^15^

Individuals who use cannabis are at an elevated risk of experiencing vessel ruptures, a condition exacerbated by the increased release of cerebral dopamine. This increase can stimulate cerebral blood flow, lead to vasospasms, and cause vasoconstriction.^39^ However, comprehensive data on the association of cannabis use with other CVDs is still lacking. There is some evidence suggesting an association between cannabis use and a heightened risk of heart failure^40^ and atherosclerosis, particularly among current tobacco users.^41^ Additionally, cannabis use has been associated with an increased risk of mortality following myocardial infarction.^42,43,44^

One study found a correlation between cannabis use and a higher risk of CVDs in individuals who do not use tobacco.^45^ Further research, even after adjusting for tobacco use, has indicated a causal relationship between cannabis use and an increased risk of small vessel strokes.^46^ Cannabis use may directly impact the CV system by raising blood pressure and heart rate, inducing vasoconstriction, and increasing carboxyhemoglobin levels, all of which elevate the risk of ischemia.^36,42^ Chronic cannabis use has been associated with higher levels of apolipoprotein C3 in the blood, a known risk factor for CVDs.^36^ Additionally, cannabis is often consumed alongside tobacco, alcohol, and other illicit drugs,^43,47^ a combination that can further amplify the risk of CV events and diseases.^46^

Sex differences for actions of cannabis use were observed in this study, which found a significant association between heavy cannabis use and CVD mortality among females overall and females who currently used tobacco but not among males. Earlier research highlighted variations by sex in both the frequency of cannabis use and the CV reactions associated with it.^4,8^ Insights from animal studies suggest that gonadal hormones might play a role in how cannabinoids affect metabolic balance and potentially influence the density of cannabinoid receptors in a sex-specific manner.^48^ The sensitivity of these cannabinoid receptors appears to be differentially affected by estrogen and testosterone, which could account for the observed sex disparities. Additionally, there is evidence indicating that over similar periods of cannabis use, females tend to consume fewer cannabis cigarettes than males.^49^ This leads to lower tetrahydrocannabinol concentrations in their bloodstream, possibly due to varying responses to the strength of the cannabis, which results in different use patterns between sexes.^50^

As this study observed for males and females overall, 1 previous study investigated the association between cannabis use and cancer mortality but did not find an association.^16^ The relationship between cannabis use and the incidence of cancer remains unclear. Current evidence, which is somewhat limited, suggests there might be an association between cannabis use and certain types of cancers, such as testicular^51^ and lung^52^ cancers, but is ambiguous for head, neck, and esophageal cancers.^16^ Future studies in humans are required to investigate the relationship between cannabis use and cancer mortality.

### Strengths and Limitations

This study’s primary strength lies in the expansive sample size provided by the UK Biobank cohort. However, its cross-sectional design introduces limitations in establishing causality, leaving open the possibility of reverse causation. A notable concern is the low response rate of 5.5% in the UK Biobank study,^53^ which could introduce participant bias. Despite this, the robust sample size^54^ and high internal validity make it unlikely that these limitations significantly influenced the observed associations.^55,56^ The study’s focus on middle-aged participants from the UK also limits the generalizability of its findings to other age groups and ethnicities. Nonetheless, the use of standardized protocols in data collection by the UK Biobank enhances the external validity of the results, ensuring consistent data collection across various conditions and personnel.

The study, however, is not without limitations. Socioeconomic data, medical history, and comorbidities were primarily gathered through self-reported questionnaires or assessments during medical examinations at health centers. Additionally, the data collection period (2006-2010) may not accurately reflect current patterns and risks associated with cannabis use. Cannabis use was self-reported rather than verified through urine or blood tests, though the reliability of self-reported cannabis use is estimated to be congruent with drug tests in 89.8% of cases.^57^ In this study, there was no information on the frequency of cannabis use in the 30 days preceding the interview, making it challenging to differentiate between associations of short-term and long-term cannabis use with mortality. The study also lacked specific data on tetrahydrocannabinol levels, cannabidiol content, and the method of cannabis consumption (eg, vaping, oral). This absence of detailed information represents a significant limitation and points to the need for further research in these areas. No information during follow-up was collected for cannabis use; thus, some participants may have continued as long-term users or not. Consequently, it is possible that the impact of prolonged, regular cannabis use was not fully captured in these findings. Future research using more precise measurements of cannabis use over extended periods is necessary to validate these results. Additionally, as the precise dosage of cannabis was not consistently recorded across all study phases, the investigation was unable to determine whether there is a dose-response relationship between cannabis use and mortality. This aspect presents an important direction for exploration in upcoming studies.

## Conclusions

This cohort study found a positive association between CVD mortality and heavy lifetime cannabis use among females. Longitudinal studies are needed in general populations to investigate potential causal effects of cannabis on mortality. Individuals using cannabis should be considered for appropriate CV risk–reduction strategies, especially among females.

## Notes

Supplement 1. **eTable 1.** Cox Proportional Hazards Regression Among Males for the Association With All-Cause Mortality **eTable 2.** Cox Proportional Hazards Regression Among Females for the Association With All-Cause Mortality **eTable 3.** Fully Adjusted Sex-Stratified Cox Proportional Hazards Regression Models for All-Cause, CVD, and Cancer Mortality According to BMI Level **eTable 4.** Fully Adjusted Sex-Stratified Cox Proportional Hazards Regression Models for All-Cause, CVD, and Cancer Mortality According to Hypertension Status **eTable 5.** Fully Adjusted Sex-Stratified Cox Proportional Hazards Regression Models for All-Cause, CVD, and Cancer Mortality According to Diabetes Status

Supplement 2.
Data Sharing Statement
