# Supplementary material for: Heavy Lifetime Cannabis Use and Mortality by Sex
Source: JAMA Netw Open. 2024 Jun 6;7(6):e2415227. doi: 10.1001/jamanetworkopen.2024.15227 (PMC11157356; doi:10.1001/jamanetworkopen.2024.15227)
Supplement: Supplement 1. — eTable 1. Cox Proportional Hazards Regression Among Males for the Association With All-Cause Mortality eTable 2. Cox Proportional Hazards Regression Among Females for the Association With All-Cause Mortality eTable 3. Fully Adjusted Sex-Stratified Cox Proportional Hazards Regression Models for All-Cause, CVD, and Cancer Mortality According to BMI Level eTable 4. Fully Adjusted Sex-Stratified Cox Proportional Hazards Regression Models for All-Cause, CVD, and Cancer Mortality According to Hypertension Status eTable 5. Fully Adjusted Sex-Stratified Cox Proportional Hazards Regression Models for All-Cause, CVD, and Cancer Mortality According to Diabetes Status [file jamanetwopen-e2415227-s001.pdf]

Supplementary Online Content

Vallée A. Heavy lifetime cannabis use and mortality by sex. *JAMA Netw Open*. 2024;7(6):e2415227. doi:10.1001/jamanetworkopen.2024.15227

eTable 1. Cox Proportional Hazards Regression Among Males for the Association With All-Cause Mortality
eTable 2. Cox Proportional Hazards Regression Among Females for the Association With All-Cause Mortality
eTable 3. Fully Adjusted Sex-Stratified Cox Proportional Hazards Regression Models for All-Cause, CVD, and Cancer Mortality According to BMI Level
eTable 4. Fully Adjusted Sex-Stratified Cox Proportional Hazards Regression Models for All-Cause, CVD, and Cancer Mortality According to Hypertension Status
eTable 5. Fully Adjusted Sex-Stratified Cox Proportional Hazards Regression Models for All-Cause, CVD, and Cancer Mortality According to Diabetes Status

This supplementary material has been provided by the authors to give readers additional information about their work.

© 2024 Vallée A. *JAMA Network Open*.

eTable 1: Cox proportional hazards regression among males for the association with all-cause mortality

Parameters	Univariable HR	Multivariable HR
Age	1.11 [1.10-1.12]	1.10 [1.09 – 1.11]
CV disease	2.71 [2.33-3.16]	1.38 [1.16 – 1.65]
BMI		
High	1.64 [1.41-1.89]	1.24 [1.05 – 1.47]
Moderate	1.12 [0.98 – 1.27]	0.96 [0.83 – 1.12]
Low	Ref.	Ref.
Income		
High	0.31 [0.27 – 0.37]	0.65 [0.54 – 0.79]
Moderate	0.57 [0.50 – 0.66]	0.73 [0.63 – 0.86]
Low	Ref.	Ref.
Education		
High	0.67 [0.57 – 0.79]	1.11 [0.91 – 1.34]
Moderate	0.82 [0.70 – 0.97]	1.08 [0.90 – 1.29]
Low	Ref.	Ref.
Alcohol		
Current	0.69 [0.50 - 0.97]	0.74 [0.52 – 1.05]
Past	1.27 [0.84 – 1.92]	1.11 [0.72 – 1.73]
Never	Ref.	Ref.
Smoking Pack years	1.019 [1.017 – 1.022]	1.010 [1.008 – 1.013]
Hypertension	1.85 [1.65 – 2.07]	1.18 [1.03 – 1.34]
Diabetes	2.28 [1.96 – 2.66]	1.44 [1.21 – 1.72]
Dyslipidemia	1.45 [1.29 – 1.63]	1.00 [0.86 – 1.14]
Antidepressant	1.61 [1.29 – 2.01]	1.41 [1.11 – 1.79]
Cannabis		
Heavy	0.71 [0.52 – 0.96]	1.28 [0.90 – 1.81]
Moderate	0.59 [0.44 – 0.79]	0.97 [0.70 – 1.36]
Low	0.64 [0.55 – 0.76]	0.88 [0.72 – 1.07]
Never	Ref.	Ref.

© 2024 Vallée A. *JAMA Network Open*.

eTable 2: Cox proportional hazards regression among females for the association with all-cause mortality

Parameters	Univariable HR	Multivariable HR
Age	1.09 [1.08-1.10]	1.08 [1.07 – 1.09]
CV disease	2.29 [1.66 – 3.17]	0.97 [0.66 – 1.42]
BMI		
High	1.61 [1.38-1.89]	1.11 [0.92 – 1.33]
Moderate	1.05 [0.91 – 1.22]	0.82 [0.70 – 0.97]
Low	Ref.	Ref.
Income		
High	0.45 [0.37 – 0.54]	0.87 [0.69 – 1.09]
Moderate	0.64 [0.55 – 0.75]	0.89 [0.75 – 1.05]
Low	Ref.	Ref.
Education		
High	0.78 [0.63 – 0.95]	1.19 [0.94 1.51]
Moderate	0.79 [0.64 – 0.97]	1.00 [0.80 – 1.26]
Low	Ref.	Ref.
Alcohol		
Current	0.58 [0.44 – 0.77]	0.71 [0.53 – 0.96]
Past	1.04 [0.70 - 1.56]	0.94 [0.61 – 1.46]
Never	Ref.	Ref.
Smoking Pack years	1.025 [1.020 – 1.029]	1.017 [1.013 – 1.022]
Hypertension	1.81 [1.59 – 206]	1.17 [1.01 – 1.35]
Diabetes	2.27 [1.81 – 2.85]	1.61 [1.26 – 2.07]
Dyslipidemia	1.81 [1.59 – 2.06]	1.17 [1.00 – 1.35]
Antidepressant	1.26 [1.01 – 1.56]	1.19 [0.94 – 1.51]
Cannabis		
Heavy	1.11 [0.72 – 1.71]	1.49 [0.92 – 2.40]
Moderate	0.77 [0.53 – 1.09]	1.07 [0.72 – 1.60]
Low	0.83 [0.69 – 1.01]	1.07 [0.87 – 1.33]
Never	Ref.	Ref.

© 2024 Vallée A. *JAMA Network Open*.

eTable 3: Fully adjusted sex-stratified Cox proportional hazards regression models for all-cause, CVD and cancer mortality according to the status of BMI level.

Males			
	All-cause mortality	CVD mortality	Cancer mortality
Obese			
Cannabis use	HR 95% CI	HR 95% CI	HR 95% CI
Heavy	1.51 [0.83 – 2.73]	0.52 [0.07 – 3.81]	0.87 [0.27 – 2.76]
Moderate	1.15 [0.64 – 2.05]	0.36 [0.06 – 2.63]	1.72 [0.86 – 3.45]
Low	1.09 [0.71 – 1.68]	0.55 [0.24 – 1.28]	1.17 [0.74 – 1.83]
Never	Ref.	Ref.	Ref.
overweight			
Cannabis use	HR 95% CI	HR 95% CI	HR 95% CI
Heavy	1.25 [0.75 – 2.08]	1.06 [0.32 – 3.43]	0.88 [0.39 – 2.02]
Moderate	0.78 [0.46 – 1.30]	0.47 [0.11 – 1.91]	0.85 [0.44 – 1.69]
Low	0.95 [0.72 – 1.25]	0.86 [0.48 – 1.52]	0.70 [0.46 – 1.05]
Never	Ref.	Ref.	Ref.
Normal BMI			
Cannabis use	HR 95% CI	HR 95% CI	HR 95% CI
Heavy	0.85 [0.21 – 3.43]	1.18 [0.27 – 5.20]	1.09 [0.46 – 2.58]
Moderate	0.85 [0.40 – 1.80]	1.99 [0.66 – 5.97]	0.82 [0.33 – 2.06]
Low	0.83 [0.43 – 1.91]	0.78 [0.30 – 2.03]	1.17 [0.74 – 1.84]
Never	Ref.	Ref.	Ref.
Females			
	All-cause mortality	CVD mortality	Cancer mortality
obese			
Cannabis use	HR 95% CI	HR 95% CI	HR 95% CI
Heavy	0.79 [0.19 – 3.21]	2.82 [0.36 – 18.38]	0.67 [0.09 – 4.91]
Moderate	1.35 [0.65 – 2.81]	3.35 [0.94 – 11.89]	1.14 [0.40 – 3.16]
Low	1.06 [0.69 – 1.64]	0.56 [0.13 – 2.45]	1.32 [0.78 – 2.20]
Never	Ref.	Ref.	Ref.
overweight			
Cannabis use	HR 95% CI	HR 95% CI	HR 95% CI
**Heavy**	**2.23 [1.11 – 4.45]**	2.74 [0.34 – 16.90]	**2.79 [1.32 – 5.88]**
Moderate	1.12 [0.54 – 2.30]	3.40 [0.74 – 15.63]	0.59 [0.18 – 1.87]
Low	1.05 [0.72 – 1.54]	1.39 [0.46 – 4.19]	1.18 [0.77 – 1.84]
Never	Ref.	Ref.	Ref.
Normal BMI			
Cannabis use	HR 95% CI	HR 95% CI	HR 95% CI
Heavy	1.19 [0.55 – 2.55]	1.53 [0.20 – 11.72]	1.01 [0.37 – 2.77]
Moderate	1.09 [0.78 – 1.51]	1.85 [0.42 – 8.17]	1.05 [0.51 – 2.17]
Low	0.89 [0.47 – 1.71]	0.96 [0.32 – 2.81]	1.14 [0.77 – 1.68]
Never	Ref.	Ref.	Ref.

© 2024 Vallée A. *JAMA Network Open*.

eTable 4: Fully adjusted sex-stratified Cox proportional hazards regression models for all-cause, CVD, and cancer mortality according to hypertension status.

Males			
	All-cause mortality	CVD mortality	Cancer mortality
Hypertension			
Cannabis use	HR 95% CI	HR 95% CI	HR 95% CI
Heavy	**1.52 [1.01 – 2.32]**	1.38 [0.60 – 3.18]	1.21 [0.61 – 2.39]
Moderate	1.11 [0.73 – 1.66]	0.91 [0.40 – 2.10]	1.17 [0.66 – 2.07]
Low	0.95 [0.76 – 1.19]	0.73 [0.45 – 1.19]	1.11 [0.82 – 1.51]
Never	Ref.	Ref.	Ref.
no Hypertension			
Cannabis use	HR 95% CI	HR 95% CI	HR 95% CI
Heavy	0.92 [0.50 – 1.72]	-	0.88 [0.39 – 2.02]
Moderate	0.76 [0.42 – 1.36]	0.45 [0.06 – 3.32]	0.85 [0.44 – 1.69]
Low	0.75 [0.53 – 1.06]	0.78 [0.32 – 1.88]	0.70 [0.46 – 1.05]
Never	Ref.	Ref.	Ref.
Females			
	All-cause mortality	CVD mortality	Cancer mortality
Hypertension			
Cannabis use	HR 95% CI	HR 95% CI	HR 95% CI
Heavy	0.24 [0.03 – 1.74]	1.60 [0.21 – 11.94]	-
Moderate	1.09 [0.56 – 2.15]	2.65 [0.79 – 8.86]	0.58 [0.19 - 1.86]
Low	1.04 [0.74 – 1.48]	1.29 [0.57 – 2.94]	1.13 [0.73 – 1.72]
Never	Ref.	Ref.	Ref.
no Hypertension			
Cannabis use	HR 95% CI	HR 95% CI	HR 95% CI
Heavy	**2.14 [1.29 – 3.54]**	2.71 [0.61 – 12.17]	**2.43 [1.36 – 4.32]**
Moderate	1.06 [0.65 – 1.76]	2.67 [0.88 – 8.11]	1.11 [0.61 – 2.01]
Low	1.09 [0.83 – 1.44]	0.56 [0.17 – 1.91]	1.26 [0.92 – 1.73]
Never	Ref.	Ref.	Ref.

© 2024 Vallée A. *JAMA Network Open*.

eTable 5: Fully adjusted sex-stratified Cox proportional hazards regression models for all-cause, CVD and cancer mortality according to diabetes status.

Males			
	All-cause mortality	CVD mortality	Cancer mortality
Diabetes			
Cannabis use	HR 95% CI	HR 95% CI	HR 95% CI
Heavy	1.42 [0.57 – 3.57]	1.78 [0.40 – 7.87]	1.63 [0.38 – 6.99]
Moderate	0.85 [0.27 – 2.71]	0.91 [0.12 – 6.88]	-
Low	1.10 [0.68 – 1.79]	0.56 [0.17 – 1.85]	1.24 [0.60 – 2.56]
Never	Ref.	Ref.	Ref.
no Diabetes			
Cannabis use	HR 95% CI	HR 95% CI	HR 95% CI
Heavy	1.21 [0.83 – 1.77]	0.76 [0.28 - 3.09]	0.93 [0.53 – 1.65]
Moderate	0.97 [0.68 – 1.37]	0.78 [0.34 – 1.79]	1.15 [0.75 – 1.77]
Low	0.83 [0.68 – 1.03]	0.77 [0.49 – 1.21]	0.90 [0.79 – 1.19]
Never	Ref.	Ref.	Ref.
Females			
	All-cause mortality	CVD mortality	Cancer mortality
Diabetes			
Cannabis use	HR 95% CI	HR 95% CI	HR 95% CI
Heavy	2.20 [0.49 – 9.93]	4.68 [0.46 – 17.74]	2.69 [0.32 – 10.92]
Moderate	0.88 [0.21 – 3.73]	1.88 [0.23 – 15.41]	1.01 [0.13 – 7.80]
Low	0.95 [0.43 – 2.16]	-	1.57 [0.57 – 4.30]
Never	Ref.	Ref.	Ref.
no Diabetes			
Cannabis use	HR 95% CI	HR 95% CI	HR 95% CI
Heavy	1.42 [0.86 – 2.37]	**2.92 [1.21 – 7.05]**	1.56 [0.87 – 2.81]
Moderate	1.09 [0.72 – 1.65]	1.75 [0.42 – 6.47]	0.92 [0.53 – 1.58]
Low	1.08 [0.87 – 1.36]	1.17 [0.59 – 2.33]	1.18 [0.90 – 1.53]
Never	Ref.	Ref.	Ref.

© 2024 Vallée A. *JAMA Network Open*.
