# Supplementary material for: Heavy Lifetime Cannabis Use and Mortality by Sex
Source: JAMA Netw Open. 2024 Jun 6;7(6):e2415227. doi: 10.1001/jamanetworkopen.2024.15227 (PMC11157356; doi:10.1001/jamanetworkopen.2024.15227)
Supplement: Supplement 2. — Data Sharing Statement [file jamanetwopen-e2415227-s002.pdf]

Data Sharing Statement

Vallée. Heavy Lifetime Cannabis Use and Mortality by Sex. *JAMA Netw Open*. Published June 06, 2024. doi:10.1001/jamanetworkopen.2024.15227

Data
Data available: No

Additional Information
Explanation for why data not available: UK Biobank data are available through the UK Biobank Access Management System (UK Biobank Access Management System: http://www.ukbiobank.ac.uk/register-apply/).
